# Efficacy of electroacupuncture treatment for generalized anxiety disorder related insomnia: a study protocol for randomized controlled trial

**DOI:** 10.3389/fpsyt.2025.1532001

**Published:** 2025-05-09

**Authors:** Wang Yao, Xi Wang, Huanyi Liu, Lumin Liu, Qian Fan, Ping Yin, Yuelai Chen

**Affiliations:** Sleep Medicine Centre, LongHua Hospital Shanghai University of Traditional Chinese Medicine, Shanghai, China

**Keywords:** generalized anxiety disorder, insomnia, electroacupuncture, randomized controlled trial, protocol

## Abstract

**Background:**

Generalized anxiety disorder (GAD) is a prevalent mental disorder characterized by excessive tension, worry, fear, and autonomic symptoms, which brings huge suffering to patients. Insomnia, one of the most common symptoms experienced by individuals with GAD, often exacerbates anxiety symptoms. Electroacupuncture (EA), a non-pharmacological treatment for insomnia, presents several advantages, including significant efficacy, minimal side effects, and high patient acceptance. However, there is a lack of high-quality randomized controlled trials evaluating the efficacy and safety of EA.

**Methods:**

This study was designed as a randomized, sham-controlled clinical trial. 84 eligible patients with GAD-related insomnia will be randomly assigned to receive either three sessions of EA or sham EA weekly for 8 weeks. The primary outcome will be the change in the Pittsburgh Sleep Quality Index (PSQI) score at week 8. Hamilton Anxiety Rating Scale (HAMA), Insomnia Severity Index (ISI), sleep diary entries, actigraphy sleep assessment, 12-item Short-Form Health Survey, and weekly usage of medication will provide a thorough evaluation of sleep, anxiety, and overall living conditions.

**Discussion:**

This study aims to evaluate the efficacy and safety of EA for treating insomnia in patients with GAD, proving EA can enhance patients’ quality of life and reduce their dependence on medications.

**Clinical trial registration:**

ClinicalTrials.gov, identifier ChiCTR2400083326.

## 1 Introduction

The essential feature of generalized anxiety disorder (GAD) is excessive anxiety and worry (apprehensive expectation) regarding various events or activities lasting for at least six months (1). The intensity, duration, or frequency of the anxiety and worry is disproportionate to the actual probability or consequences of the anticipated event, significantly disrupting with psychosocial functioning (2). The 12-month prevalence of GAD is 0.9% among adolescents and 2.9% among adults in the general community of the United States (3). In urban China, the prevalence of undiagnosed or diagnosed GAD is 5.3%, with only 0.5% receiving a diagnosis (4). Insomnia is a common clinical condition characterized by difficulty initiating or maintaining sleep (5) and is also a prevalent comorbid symptom in individuals with mental health disorders (6). Many diseases can lead to cognitive deficits and affect mood (7, 8). The etiology of mood disorders and insomnia is multifactorial, with research indicating that distinct personality traits may contribute to a bidirectional interplay with mental disorders (9). Studies have substantiated that sleep disturbances exacerbate negative emotions (10, 11), while mental disorders develop bad habits (12). The survey suggests that individuals suffering from insomnia are fivefold more susceptible to anxiety or depression than those without the condition (13), while approximately half of individuals with GAD report difficulties in initiating sleep, maintaining sleep, or experiencing early morning awakenings (14).

Contemporary medical treatment for GAD-related insomnia primarily encompasses psychotherapy, pharmacotherapy, and physical therapy (15). According to clinical guidelines, evidence supports the benefits of certain types of pharmacotherapy, psychotherapy, or a combination of both for GAD (16). Cognitive Behavioral Therapy (CBT)-guided psychotherapy typically relies on a personalized treatment approach. Several countries have now implemented internet-delivered cognitive behavioral therapy, which has demonstrated efficacy in alleviating mood disorders among patients (17), however, adherence among Chinese patients often falls short of anticipated levels (18). In the context of pharmacological interventions, certain medications demonstrate favorable response and remission rates, as well as good tolerability when used to treat generalized anxiety disorder (GAD), but the effectiveness is not certain due to the relatively few comparative studies (19). Additionally, the variable spectrum of toxicity and side effects of these medications during the initial treatment phase significantly constrain their clinical application (20). Long-term benzodiazepine administration is associated with adverse reactions, increasing the risk of medication tolerance and dependence (21). Consequently, existing therapeutic approaches for generalized anxiety disorder (GAD) accompanied by insomnia frequently fall short of patient expectations.

Acupuncture presents several advantages in treating GAD-related insomnia, including considerable efficacy, convenient operation, minimal side effects, and lack of addictive properties, all of which can enhance patient treatment adherence. In this study, the primary focus was on observing changes in insomnia symptoms before and after acupuncture treatment. Therefore, the main efficacy indicator selected was the Pittsburgh Sleep Quality Index (PSQI). PSQI is used to assess the sleep quality of participants over the past month and is suitable for evaluating the sleep quality of individuals with insomnia and mental disorders (22). Most high-quality clinical studies and reviews related to GAD use the Hamilton Anxiety Scale (HAMA) to assess the level of anxiety in patients (23–25). Consequently, we used the HAMA to comprehensively evaluate both mental and physical symptoms before and after treatment (26). The Insomnia Severity Index (ISI) is a commonly used self-assessment tool for insomnia, evaluating participants’ subjective perception of the nature and symptoms of their sleep disturbances (27, 28). Sleep diaries and actigraphy combine subjective and objective recordings of patients’ sleep parameters (29, 30). The sleep diary provides necessary information for the calculation of actigraphy parameters, while the actometer overcomes recall bias that can occur when patients use sleep diaries. The 12-Item Short-Form Health Survey (SF-12) is a widely used health survey tool for assessing individuals’ health status and quality of life (31). Using the above methods, we conducted a comprehensive evaluation of generalized anxiety disorder related insomnia from both subjective and objective perspectives. Previous studies have suggested that acupuncture and electroacupuncture (EA) are appropriate, safe, and effective alternatives for managing anxiety (32). A clinical trial demonstrated that acupuncture significantly improved Hamilton Anxiety Rating Scale (HAMA) and Generalized Anxiety Disorder-7 (GAD-7) scores in comparison to sham acupuncture, verifying its effectiveness in treating GAD (33). To date, no randomized controlled trial has specifically evaluated the efficacy of electroacupuncture (EA) against sham EA for the treatment of insomnia associated with generalized anxiety disorder (GAD). Consequently, the present study employs a randomized, controlled trial design to identify an effective set of acupoints and to compare its therapeutic effects with those of sham EA.

The effective acupoint combination is derived from the long-term outpatient treatment experience of our research group. Each point works in conjunction with others to collectively reduce anxiety and calm the mind and spirit. The sleep conditions of patients with GAD will be comprehensively evaluated using subjective and objective indicators and scales. This study aims to assess the clinical efficacy and safety of EA in treating GAD-related insomnia, thereby providing substantial clinical evidence for the use of EA in this context.

## 2 Methods

### 2.1 Study design

**Figure 1:**
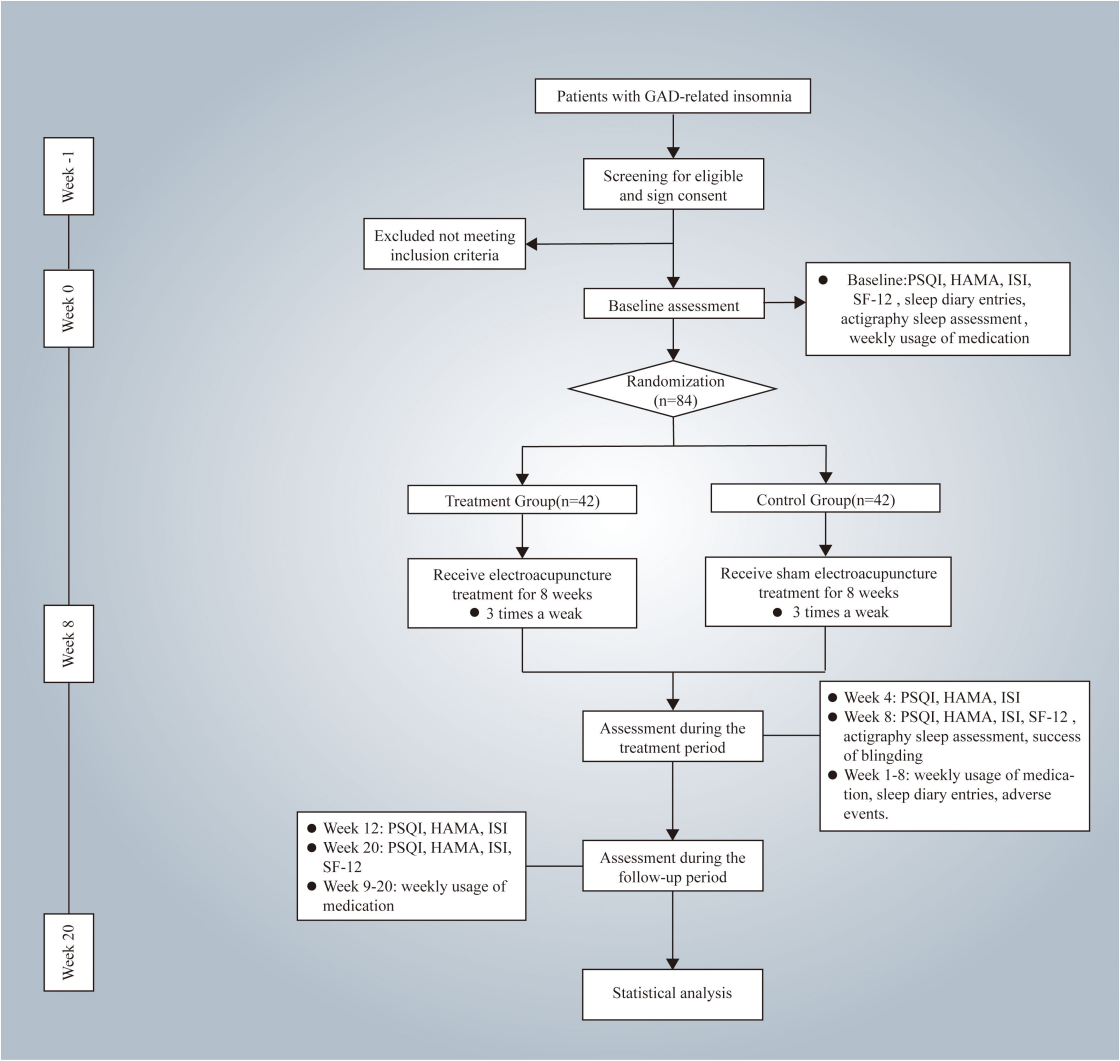
Flowchart of the study. HAMA, Hamilton Anxiety Scale; PSQI, the Pittsburgh Sleep Quality Index; ISI, Insomnia Severity Index; SF-12, 12-Item Short-Form Health Survey.

This study is a randomized, single-blinded, sham-controlled trial that has been registered with the Chinese Clinical Trial Registry (ChiCTR2400083326. The protocol adheres to the Standard Protocol Items: Recommendations for Intervention Trials (SPIRIT) 2013 (34), as detailed in supplementary file 1. Patients or the public were not involved in the design, conduct, reporting, or dissemination plans of our research. Approval for this study was approved by the Medical Ethics Committee of LongHua Hospital Shanghai University of Traditional Chinese Medicine (NO.2024LCSY020). Any modifications to the protocol will be reported to both the ChiCTR and the Medical Ethics Committee. This protocol is version 1.0. The flowchart of the study is shown in **Figure 1** (Flowchart of the study), while the schedule of enrollment, interventions, and assessment is shown in **Table 1** (Schedule of enrolment, interventions, and assessment).

**Table 1:** Schedule of enrolment, interventions, and assessments.

Study period	Enrolment	Treatment period								Follow-up period	
Time point(Week)	-1~0	1	2	3	4	5	6	7	8	12	20
Sign informed consent	x										
Eligibility screening	x										
Demographic data	x										
Medical history	x										
HAMD	x										
Primary outcomes											
PSQI	x				x				x	x	x
Secondary outcomes											
HAMA	x				x				x	x	x
ISI	x				x				x	x	x
SF-12	x								x		x
Sleep diary entries	x	x	x	x	x	x	x	x	x		
Actigraphy sleep assessment	x								x		
Weekly usage of medication	x	x	x	x	x	x	x	x	x	x	x
Adverse events		x	x	x	x	x	x	x	x		
Success of blinding									x		

### 2.2 Patient recruitment

84 patients with GAD-related insomnia will be recruited from outpatient clinics at the Sleep Medicine Center of Longhua Hospital Shanghai University of Traditional Chinese Medicine. Initially, WY, the trained personnel, will assess the eligibility of these patients through face-to-face evaluations based on the inclusion and exclusion criteria outlined below. Prior to randomization, informed consent in written form will be secured from all participants. Furthermore, all participants reserve the right to withdraw from the study at any moment, and their personal information will be strictly utilized for medical research.

### 2.3 Criteria for diagnosis

Participants meeting the following criteria will be included

According to the “*Diagnostic and statistical manual of mental disorders: DSM-5” (* 3) released by American Psychiatric Association, the diagnostic criteria for “GAD” are as follows:

1. Excessive anxiety and worry (apprehensive expectation), occurring more days than not for at least 6 months, about a number of events or activities (such as work or school performance);
2. The individual finds it difficult to control the worry. The anxiety and worry are associated with three (or more) of the following six symptoms (with at least some symptoms having been present for more days than not for the past 6 months): 1) Restlessness or feeling keyed up or on edge. 2) Being easily fatigued. 3) Difficulty concentrating or mind going blank. 4) Irritability. 5) Muscle tension. 6) Sleep disturbance (difficulty falling or staying asleep, or restless, unsatisfying sleep);
3. The anxiety, worry, or physical symptoms cause clinically significant distress or impairment in social, occupational, or other important areas of functioning;
4. The disturbance is not attributable to the physiological effects of a substance (e.g., a drug of abuse, a medication) or another medical condition (e.g., hyperthyroidism);
5. The disturbance is not better explained by another mental disorder (e.g., anxiety or worry about having panic attacks in panic disorder, negative evaluation in social anxiety disorder [social phobia], contamination or other obsessions in obsessive-compulsive disorder, separation from attachment figures in separation anxiety disorder, reminders of traumatic events in posttraumatic stress disorder, gaining weight in anorexia nervosa, physical complaints in somatic symptom disorder, perceived appearance flaws in body dysmorphic disorder, having a serious illness in illness anxiety disorder, or the content of delusional beliefs in schizophrenia or delusional disorder).

### 2.4 Criteria for inclusion

Patients will be eligible if they satisfy any of the following inclusion criteria

1. Patients met the DSM-V diagnostic criteria for GAD, accompanied by insomnia symptoms such as difficulty in falling asleep or maintaining sleep or waking up early;
2. Aged 18–75 years old. No gender limit;
3. 14 ≤ HAMA (35) score < 29 (36);
4. PSQI score (22) >10 (37);
5. Hamilton Depression Scale (HAMD-17) (38) score ≤ 17 (39) and suicide factor score of HAMD-17 ≤ 2;
6. Patients who did not change the regimen/dose of anti-anxiety drugs (escitalopram, paroxetine, venlafaxine, buspirone, duloxetine, tandospirone) or did not take any anti-anxiety drugs within the last 4 weeks before the study;
7. No mental retardation, able to understand the terms of each scale and complete the assessment;
8. Agree to participate in this study and sign the informed consent form.

### 2.5 Criteria for exclusion

Participants will be excluded if they satisfy any of the following exclusion criteria:

1. Severe heart, liver, kidney, or other major diseases;
2. Anxiety disorders caused by other physical illnesses (e.g., hyperthyroidism, pheochromocytoma, etc.);
3. Other types of anxiety disorders (e.g., social anxiety disorder, obsessive-compulsive disorder, post-traumatic stress disorder), schizophrenia spectrum and other psychotic disorders, or bipolar disorder, etc.;
4. Pregnancy or lactation period;
5. History of drug abuse or addiction;
6. Patients who changed their sedative-hypnotic medication regimen or dose within 2 weeks before the baseline visit or who received other treatments for insomnia (e.g., cognitive behavioral therapy) within 3 months before the baseline visit, which could affect the evaluation of efficacy;
7. With irregular sleep patterns or those engaged in long-term night shifts;
8. Patients who have participated in other clinical medical trials within the last 2 months;
9. With other organic sleep problems, such as sleep apnea syndrome, restless legs syndrome, etc.;
10. Contraindications for acupuncture treatment, such as having other severe life-threatening underlying diseases, or if the selected acupuncture points area has ulcers, abscesses, skin infections, limb deficiencies, etc., making it impossible to perform acupuncture treatment;
11. Patients with a cardiac pacemaker;
12. Patients with metal allergies or fear of EA.

### 2.6 Criteria for removal

1. Patients who did not complete the full course of 24 sessions over 8 weeks and did not provide a primary outcome indicator after treatment.
2. Patients with the above exclusion criteria occurring during treatment;
3. Voluntary patients’ withdrawal from study treatment;
4. Patients who change the regimen/dose of anti-anxiety drugs used in the baseline period
5. Patients who change the regimen of sedative-hypnotic medication used in the baseline period.

### 2.7 Randomization, allocation concealment and blinding

Participants in the study will be randomized into treatment and control groups in a 1:1 ratio using a random assignment scheme generated by the “Proc Plan” procedure of SAS 9.4 statistical analysis software (SAS Institute, Cary, NC, United States). This scheme will be implemented through random allocation cards, which will be sealed in opaque envelopes by independent investigators, ensuring that the envelopes and card numbers match. The independent investigators responsible for randomization reviewed the envelopes and subsequently informed the acupuncturist of the patient’s group allocation. Patients are assigned to groups according to the allocation indicated on the card within the envelope they open, based on the order of their clinic visits. Each group, the EA group and the sham EA group, comprises 42 subjects. All participants will be informed that they have an equal chance of being assigned to either the EA or sham EA group. Interventions of two groups were administered by an acupuncturist who had specific training in the field, with a minimum of 5 years of experience. Participants, information assessors, and statisticians will not be able to predict group assignments throughout the study, except for the acupuncturist. To ensure the success of the blinding, communication regarding the treatments is minimized, appointments are scheduled, and patients are treated in separate consultation rooms while lying supine and wearing eye masks. Moreover, all researchers who have received training on the implementation of this research will strictly abide by the principle of departmental separation.

### 2.8 Interventions

All participants will receive three sessions per week (every other day), each lasting for 30 minutes, resulting in a total of 24 sessions over the 8-week course. Follow-up assessments will occur at weeks 12 and 20. Participants were asked to return for follow-up visits at weeks 12 and 20.

Participants in both groups will receive sleep hygiene guidelines (e.g., avoiding coffee, tea, alcohol, etc.; maintaining a routine; creating an appropriate sleep environment; and attending to emotional self-regulation) through conversation. These instructions will serve as the primary treatment for GAD-related insomnia. The conversations will be conducted from week 0 to week 8. Additionally, participants will be instructed to maintain a sleep diary for 9 weeks, covering week 0 to 8.

During the trial, both the type and dosage of anti-anxiety medications will remain unchanged. The type of sedative-hypnotic medications will also remain consistent, however, dosage adjustments may be made based on the patient’s condition, with detailed records maintained. In cases of severe insomnia, the temporary use of Zolpidem or Eszopiclone is permitted for no more than three consecutive days with thorough documentation of such instances.

#### 2.8.1 Treatment group

**Figure 2:**
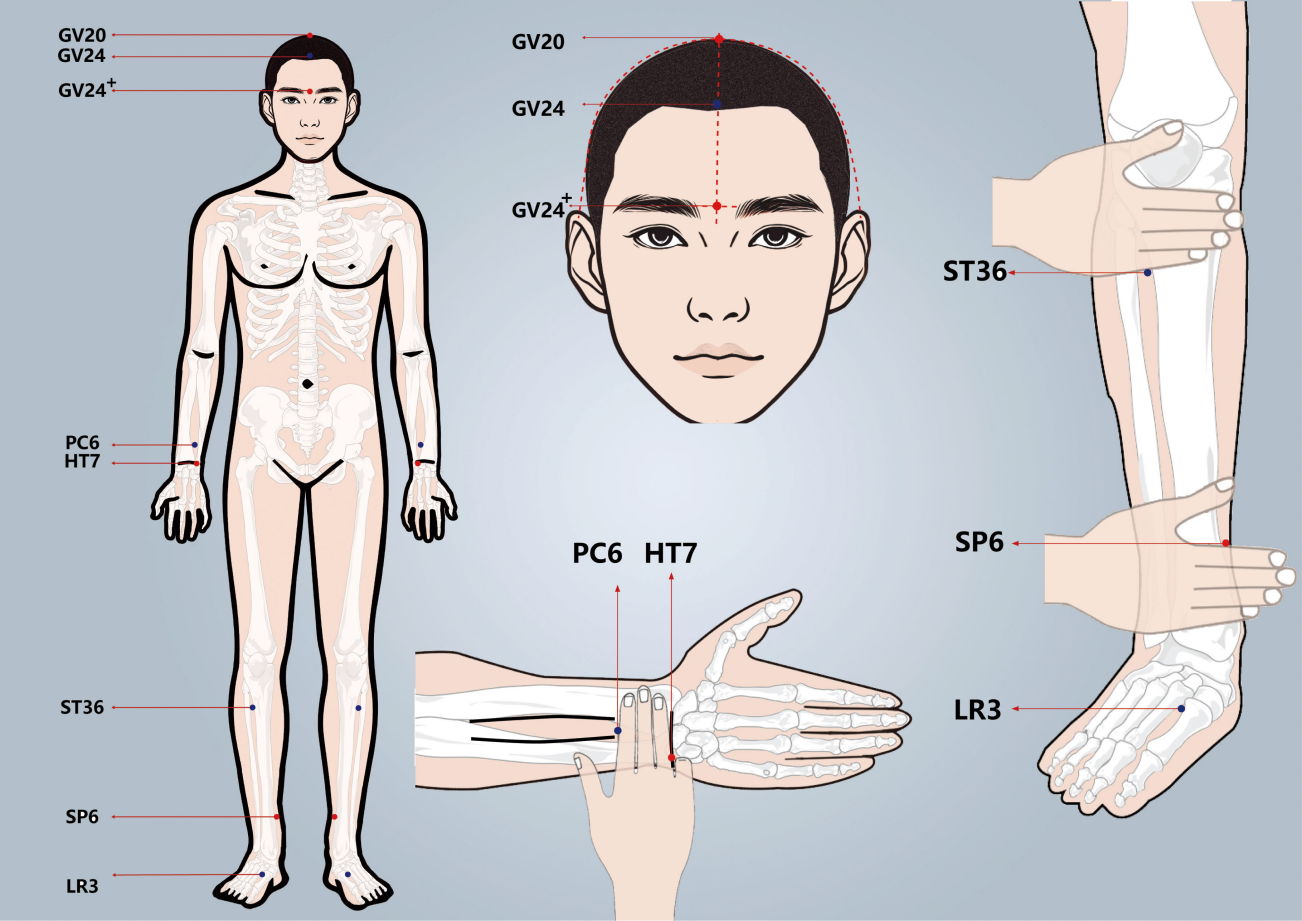
Locations of acupoints. Image of acupoints for treatment and control group.

**Figure 3:**
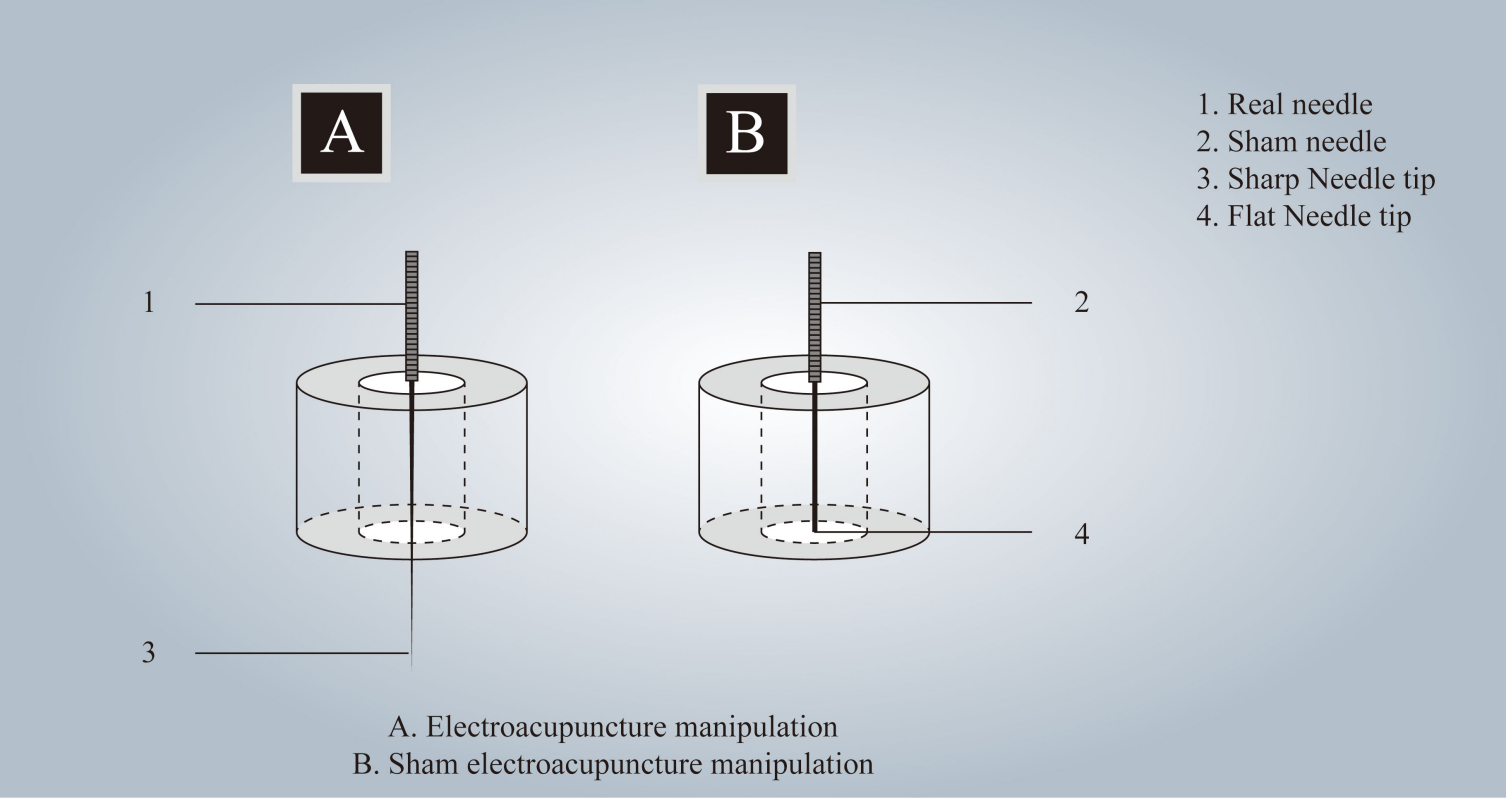
EA and sham EA manipulation. Electroacupuncture and sham Electroacupuncture manipulation.

Participants in the treatment group will receive acupuncture at Baihui(GV20), Shenting (GV24), Yintang (GV24^+^), Neiguan (PC6), Shenmen (HT7), Zusanli (ST36), Sanyinjiao (SP6), and Taichong (LR3). Among these acupoints, GV20, GV24, and GV24^+^ are located on the anterior midline, while the others are applied bilaterally (**Figure 2** Locations of acupoints). Acupoint Location: refer to *The Location of Acupoints: State Standard of the People’s Republic of China* [GB/T 12346–2021]). The acupuncture technique for each point is described in **Table 2** (Locations and manipulations of acupoints in the treatment group). For the treatment group, patches will be attached to the acupoint (**Figure 3** EA and sham EA manipulation). Needles will be inserted through the patches into the skin, and all needles will undergo acupuncture manipulation such as lifting, twirling, and thrusting to achieve “*De qi*”, which is characterized by sensations of swelling, soreness, numbness, and heaviness. GV20 and GV24^+^ will be connected to EA with a continuous wave of 2 Hz (40) and an intensity adjusted to the patient’s comfort level, lasting for 30 minutes. In this study, sterile acupuncture needles (size 0.25*25mm and 0.25*40mm from Suzhou Medical Supplies Factory Co.’s Hwato) and an electrical stimulator (SDZ-V EA instrument, Hwato, Suzhou, China) will be utilized.

**Table 2:** Locations and manipulations of acupoints in the treatment group.

Acupoint	Location	Manipulation
Baihui (GV20)	On the head, 5 *cun* superior to the anterior hairline, on the anterior median line.	Insert the needle transversely for 0.5-0.8 *cun*, and connect to EA with a continuous wave of 2Hz.
Shenting (GV24)	On the head, 0.5 *cun* superior to the anterior hairline, on the anterior median line.	Insert the needle transversely for 0.5-0.8 *cun*.
Yintang (GV24^+^)	On the forehead, at the midpoint between the medial ends of the eyebrows.	Insert the needle transversely for 0.3-0.5 *cun*, and connect to EA with a continuous wave of 2Hz.
Neiguan (PC6)	On the anterior aspect of the forearm, 2 *cun* superior to the palmar wrist crease, between the palmaris longus tendon and the flexor carpi radialis tendon.	Insert the needle perpendicularly for 0.5-1.0 *cun*.
Shenmen (HT7)	On the anterior aspect of the wrist, at the ulnar end of the palmar wrist crease, radial to the flexor carpi ulnaris tendon.	Insert the needle perpendicularly for 0.3-0.5 *cun.*
Zusanli (ST36)	On the lateral aspect of the leg, 3 *cun* distal to the lower border of the patella and 1 finger-breadth lateral to the anterior crest of the tibia.	Insert the needle perpendicularly for 1.0-1.5 *cun.*
Sanyinjiao (SP6)	On the medial aspect of the leg, 3 *cun* superior to the rominence of the medial malleolus, posterior to the medial border of the tibia.	Insert the needle perpendicularly for 1.0-1.5 *cun.*
Taichong (LR3)	On the dorsum of the foot, between the first and second metatarsal bones, in the depression anterior to the junction of the bases of the two bones, or over the dorsalis pedis artery.	Insert the needle perpendicularly for 0.5-1.0 *cun.*

#### 2.8.2 Control group

The acupoints selected for patients in the control group are identical to those used in the treatment group. Patches are affixed to the acupoints (**Figure 3** EA and sham EA manipulation), allowing sham needles to be inserted through the patch and reach the surface of the skin. Because of the flat needle tip, it cannot penetrate the skin (41, 42) (Patent Number: ZL 2023 2 0605629.3). GV20 and GV24^+^ are connected to the same type of electrical stimulator via a special power cord, the internal wires of which have been cut and are not visible externally, lasting for 30 minutes. As a result, the EA device appears to be connected, but no current flows through the wires. In this group, the sham needles (size 0.25*25mm from Guizhou Andi Medical Equipment Co.) and the same electrical stimulator will be utilized.

### 2.9 Outcome

#### 2.9.1 Primary outcome

The primary outcome measure is the change in the Pittsburgh Sleep Quality Index (PSQI) score at week 8, compared to baseline.

PSQI is used to assess the sleep quality of subjects over the past month. It is applicable for the evaluation of sleep quality in patients with sleep disorders. The PSQI consists of 19 questions divided into 7 dimensions, including sleep quality, sleep efficiency, nocturnal activities, early waking, sleep medication, sleep satisfaction, and daytime fatigue. Due to its simplicity, high reliability, and validity, and its high correlation with polysomnographic results, it has become a commonly used scale in research and psychiatric clinical assessments (22).

#### 2.9.2 Secondary outcome

The secondary outcomes include the following seven items:

1. The change in HAMA score compared to the baseline. The Hamilton Anxiety Rating Scale (HAMA), one of the first rating scales to measure the severity of perceived anxiety symptoms, is still in use today and is considered one of the most widely used rating scales. It can effectively compare changes in symptoms before and after treatment to assess the effectiveness of the treatment (26). It will be assessed at weeks 4, 8, 12, and 20.
2. The change in PSQI score compared to the baseline will be evaluated at weeks 4, 12, and 20.
3. The change in Insomnia Severity Index (ISI) score relative to the baseline. It will be evaluated at weeks 4, 8, 12, and 20.
4. Sleep diary entries: Records subjective estimates daily from week 0 to week 8. The recording includes Sleep Onset Latency, Wake After Sleep Onset, Early Morning Awakening, Total Sleep Time, and Sleep Efficiency.
5. The change in the 12-item Short-Form Health Survey (SF-12) score compared to the baseline, which will be evaluated at weeks 8 and 20.
6. Actigraphy sleep assessment: Patients will wear an actigraphy motion recorder for four days during weeks 0 and 8. The recordings will include total sleep time, sleep awakening numbers, wake after sleep onset, average duration of awakening, and sleep efficiency. This device will objectively assess the subjects’ sleep quality.
7. Weekly usage of medication: The usage of sedative-hypnotic medication will be recorded for each group of subjects weekly from week 0 to 20. Adjustments to sedative-hypnotic medication during the treatment and follow-up period will be documented strictly, including the name, dosage, and frequency of the medication.

### 2.10 Blinding assessment

After participants complete their final treatment, an independent assessor will present them with three options: EA, sham EA, and uncertainty. Their selections will be documented, and the results will be used to assess the success of the blinding process.

### 2.11 Safety evaluation

Treatment-related adverse events (AEs) include dizziness, fainting, hematoma, needle breakage, infection, and exacerbation of the condition due to excessive stimulation from acupuncture or EA. Each AE will be documented with details regarding the date of occurrence, severity, relevance, and resolution. Prior to the trial, researchers will undergo a rigorous training program to ensure that they are proficient in both the treatments and the management of AEs. In the event of any observed AEs, the acupuncturist will promptly assess the severity and take appropriate actions. These may include immediate needle removal and cessation of treatment, providing rest, applying gentle pressure to mitigate bleeding and hematoma formation, or referring the patient to a surgeon or other medical professional in cases of needle breakage or infection. Serious AEs will be reported to the safety committee without delay and managed actively. The anxiety level of the participants was evaluated by a trained assessor, who will terminate the trial based on the outcome. Finally, researchers will analyze patient case report form (CRF) data on AEs to evaluate the occurrence of adverse events in this trial, thereby providing valuable insights for the design of future trials.

### 2.12 Sample size calculation

The PASS software (version 15.0.5, NCSS, LLC) was used to calculate sample size with α of 0.05 (two-tailed), power of 80%, and ratio of 1:1. Based on our pilot study, the change in PSQI score of patients with GAD-related insomnia between week 8 and baseline was calculated. For the treatment and control groups, the mean ± standard deviation (SD) was 6.24 ± 3.63 and 3.69 ± 2.45, respectively. The calculated sample size for each group is 33 cases; considering a 20% dropout rate, each group needs 42 participants, totaling 84 participants for both groups.

### 2.13 Statistical methods

Statistical analysis, including a full analysis set based on the intent-to-treat principle and a per-protocol set, will be conducted with SPSS 26.0 software. Continuous variables such as the change in PSQI, HAMA, ISI, SF-12 score compared to the baseline and indicators of sleep diary entries and actigraphy sleep assessment, which is described as mean ± SD or median (interquartile range) will be analyzed by t-test or rank-sum test while categorical variables such as the proportion of patients with reduced medication represented by frequency (composition ratio) will be analyzed by chi-square or Fisher’s exact tests. CIs will be set at 95% with a significance level of 5% (P < 0.05). The success of blinding will be evaluated through Bang’s Index by using the “χ² test” procedure of SPSS 26.0 software. In addition, A subgroup analysis will be conducted based on factors such as age, gender, type of anti-anxiety medication, and type of sedative-hypnotic medication by the “Split File” procedure of SPSS 26.0 software. The confidence interval will be set at 95%, with a significance level of 5% (P < 0.05).

### 2.14 Quality control, data management, and monitoring

Before treatment, a licensed acupuncturist with a minimum of 5 years’ clinical experience will receive rigorous standardized training in acupoint locations, manipulation techniques, using EA devices, and using appropriate patches for EA and sham EA. Researchers will receive standardized training on the study protocol, standard operating procedures, and scale evaluation to enhance research validity. We designed a Case Report Form specifically for this trial, which was evaluated and recorded by one researcher, stored at the hospital. An extensive data management plan will be developed, encompassing data collection, entry, and handling. To ensure accuracy, data will be entered independently by one researcher, and then data will be confirmed by another researcher. Data quality and research progress will be checked regularly by research assistants and supervised by monitors. The monitoring committee comprises individuals well-versed in clinical practice, research methodologies, and statistics. It operates independently from the study team, maintaining no direct involvement in the trial’s execution. Their responsibilities encompass overseeing trial conduct, decision-making processes, and ensuring the integrity and safety of the gathered data every 6 months.

### 2.15 Trial status

The intended trial period is from April 2024 to February 2025. This clinical trial is now recruiting participants. The clinical trial registry will be updated with key protocol changes upon report publication. The study’s findings will be disseminated through peer-reviewed journals or presented at scholarly conferences. Patient data will be anonymized to safeguard participant confidentiality before publication.

## 3 Discussion

The symptoms of GAD accompanied by insomnia significantly impair the cognitive and social functioning of patients, imposing a substantial burden on individuals, families, and society (43). Treating GAD with co-occurring insomnia is particularly challenging, with low rates of complete remission (16). Acupuncture, as one of the most important external treatment techniques within traditional Chinese medicine (TCM), is widely applied in the treatment of insomnia and other mental disorders (42, 44, 45), with advantages such as significant efficacy, ease of operation, and few side effects. Previous research (33) demonstrated that compared to the sham acupuncture group, acupuncture can significantly improve anxiety for perimenopausal women with GAD. Another research (46) found that adding acupuncture to standard medication treatment for GAD significantly reduced HAMA and PSQI scores after four weeks of treatment, suggesting that integrating acupuncture into the treatment regimen for GAD with insomnia can significantly alleviate anxiety symptoms and improve sleep quality. Comparative study (40) on the efficacy of EA versus traditional acupuncture in treating anxiety symptoms have found that both methods rapidly reduce anxiety levels, as evidenced by reductions in Beck Anxiety Inventory, GAD scale, and overall anxiety levels, independent of anti-anxiety medication use. De qi, an essential prerequisite for optimal acupuncture efficacy (47), constitutes a multisensory phenomenon experienced jointly by patients and practitioners during needling interventions. During De qi elicitation, patients typically report numbness, dull ache, heaviness, regional pain, or distension at needling sites (48), while the acupuncturist perceives tactile feedback through increased tissue resistance around the needle (49).

However, there is a lack of high-quality randomized controlled trials (RCTs) on the effects of EA on insomnia and anxiety symptoms in patients with GAD, as well as on the efficacy during follow-up periods. To enhance the reliability of results and minimize bias, we will implement several quality control measures. First of all, both real and sham acupuncture treatments will employ an identical auxiliary device (ZL 2023 20605629.3), which is visually indistinguishable to meet blinding requirements and promote better patient compliance. Previous clinical studies (50) have validated this manipulation of sham EA as feasible. Secondly, a comprehensive evaluation of patients’ sleep quality will be conducted using both objective and subjective measures, including PSQI, ISI, sleep diaries, and actigraphy sleep assessment, which will provide a more comprehensive understanding of patients’ sleep patterns. Thirdly, the HAMA scale was utilized to evaluate anxiety levels, while the assessment of patients’ quality of life was also conducted. This comprehensive assessment will provide a thorough evaluation of sleep, anxiety, and overall living conditions in patients with GAD-related insomnia.

Nevertheless, there are limitations to the current study design. First, this trial is not conducted as a multicenter study but is limited to patients recruited from a single hospital in one city, which may raise concerns about bias due to regional factors. Moreover, given the inherent characteristics of acupuncture interventions, such as the needle passing through the skin and the technique used to elicit the “De qi” sensation, it is not feasible to implement a double-blind method. Thirdly, the mechanisms by which acupuncture improves the insomnia and anxiety symptoms of patients with GAD are not yet well understood, and further experiments are needed to uncover the underlying mechanisms.

## 4 Conclusion

The current research seeks to determine the efficacy and safety of EA for patients with GAD-related insomnia by contrasting it with sham EA. This assessment utilizes both subjective and objective indicators and scales, aiming to establish an objective, scientific rationale for clinical treatment and application.
